# QRFP43 modulates the activity of the hypothalamic-pituitary-thyroid axis in female sheep

**DOI:** 10.1038/s41598-025-85693-w

**Published:** 2025-01-07

**Authors:** Bartosz Jarosław Przybył, Michał Szlis, Anna Misztal, Anna Wójcik-Gładysz

**Affiliations:** Department of Animal Physiology, The Kielanowski Institute of Animal Physiology and Nutrition, Polish Academy of Sciences, Instytucka 3, Jabłonna, 05-110 Poland

**Keywords:** Hypothalamus, QRFP43, Sheep, Thyroid hormones, Neurophysiology, Hypothalamus, Pituitary gland, Thyroid gland

## Abstract

Since the early discovery of QRFP43, intensive research has been primarily focused on its role in the modulation of food intake. As is widely recognised, the regulation of the body’s energy status is a highly complex process involving numerous systems, hormones and neurotransmitters. Among the most important regulators of energy status, alongside the satiety and hunger centre located in the hypothalamus, is the HPT axis, which directly and indirectly affects the regulation of metabolism in all cells of the body. Therefore, it seems highly important to conduct studies aimed at elucidating how QRFP43 may impact the secretory activity of the HPT axis. The objective of this work was to investigate the role of QRFP43 in modulating HPT axis activity in sheep. The study examined mRNA and peptide expression of TRH and TSH in the hypothalamus and pituitary, as well as plasma concentrations of TSH, free T4 (FT4) and free T3 (FT3). Moreover, the relationship between QRFP34 and mRNA expression of the *Dio1*, *Dio2*, and *Dio3* genes was explored in selected tissues of the HPT axis. The animals (*n* = 48) were randomly divided into three experimental groups: a control group receiving an ICV infusion of Ringer-Locke solution, and two experimental groups receiving ICV infusions of QRFP43 at doses of 10 and 50 µg per day. Four 50-minute ICV infusions were administered to all sheep at 30 min intervals each of three consecutive days. Hypothalamic, pituitary and thyroid glands were collected and preserved for further immunohistochemical and molecular biological analyses. Additionally, blood samples were collected during the experiment for subsequent RIA determinations. In summary, the results of the experiment have indicated that QRFP43 modulates the secretory activity of the HPT axis at all organisational levels. Moreover, QRFP43 can alter the mRNA expression profiles of DIO1, DIO2 and DIO3 in HPT tissues, leading to discrete changes in the metabolism of the cells studied and their response to signals transmitted by T4 and T3.

**Supplementary Information:**

The online version contains supplementary material available at 10.1038/s41598-025-85693-w.

## Introduction

The hypothalamic-pituitary-thyroid (HPT) axis is a multi-level regulatory system responsible for maintaining physiological processes such as metabolism, maintenance of energy balance, linear bone growth, foetal brain development (myelination of neurons) or regulation of reproductive function (ovulatory cycle and spermatogenesis). Thyrotropin-releasing hormone (TRH) is the primary hormone of the HPT axis, with its synthesis predominantly occurring in the paraventricular nucleus (PVN) of the mediobasal hypothalamus (MBH)^1,2^. TRH is involved in stimulating the synthesis and release of thyrotropin (TSH) from the anterior pituitary. TSH, in turn, stimulates the production of subsequent thyroid hormones, namely thyroxine (T4) and triiodothyronine (T3), by the thyroid gland^3^. Transport of T3 and T4 throughout the body is subsequently facilitated by highly specific plasma proteins such as thyroxine-binding globulin, transthyretin, and albumin^4^.

It should be mentioned that the ratio between T3 and T4 in the body is regulated by processes affecting their conversion *via*enzymes referred to as iodothyronine deiodinase type 1 (Dio1), type 2 (Dio2), and type 3 (Dio3). Previous studies have indicated that Dio1, along with Dio2 and Dio3, plays a role in the conversion of T4 to T3. However, while Dio2 and Dio3 regulate T3 levels intracellularly, Dio1 is additionally responsible for delivering T3 into the bloodstream^5,6^. Furthermore, T4 can also be converted by Dio3 to the biologically inert reverse T3 (rT3). This mechanism acts as a ‘buffer’ to prevent excessive T3 synthesis. In addition to their downstream effects, T4, T3, and rT3 provide negative feedback at the level of the hypothalamus and pituitary to inhibit the secretion of TRH and TSH^7^.

Current knowledge indicates that the HPT axis may not exclusively be auto-regulated through short or long feedback loops, but may also be modulated through other hormones and neurotransmitters such as leptin, neuropeptide Y (NPY), agouti-related protein (AgRP), melatonin or RF-amide peptides^6,8^. RF-amide peptides are described as a family of regulatory peptides containing an arginine and amidated phenylalanine (RF; Arg-Phe-NH2) sequence at their C-terminus. In mammals, seven RF-amide peptides, encoded by five distinct genes, have been characterised to date: neuropeptide FF group, prolactin-releasing peptide, pyroglutamylated RF-amide peptide and kisspeptin^9^.

The 26RFa/QRFP43 peptide is the most recently identified member of this family, and the sequence encoding the 26RFa precursor is present in the brain area in all vertebrate phyla^10^. In mammals, cleavage of the 26RFa precursor could generate two forms of this peptide: 26RFa and QRFP43, which is the N-terminal elongated form and possesses the same biological activity as 26RFa^11,12^. It is noteworthy, that the sequence of the C-terminal domain of this peptide, which governs its biological activity, has shown strong conservation during the evolution^13^. This characteristic is indicative of a key role played by this neuropeptide in controlling vital neuroendocrine functions in vertebrate organisms. Findings from studies across various species revealed that the highest expression level of the 26RFa/QRFP43 gene was detected in the hypothalamus area in the ARC, PVN, ventromedial hypothalamus (VMH), and lateral hypothalamic area (LHA)^11,14,15^.The action of 26RFa/QRFP43 in humans is mediated by the GPR103 receptor. It is worth emphasising that high expression of GPR103 mRNA has been particularly observed in the hypothalamic and brainstem nuclei, including the VMH, dorsomedial hypothalamic nuclei, PVN, ARC, LHA and the nucleus of the solitary tract^12,16–18^.

In the available literature concerning the functioning of the HPT axis and RF-amide family peptides, the predominant focus revolves around describing the mechanisms of information transfer from the HPT axis to the hypothalamus-pituitary-gonadal (HPG) axis. As evidenced by numerous studies, melatonin emerges as the primary hormone involved in this signal transduction^6,19^. However, there is a lack of research elucidating whether RF-amides themselves can exert any effect on the HPT axis. Considering the physiological role of RF-amides in the body and the demonstrated synthesis of their active protein forms within the same hypothalamic structures where TRH is expressed and synthesised, studying their associations is crucial to understand the role of these peptides in the body.

The purpose of this study was to investigate the role of QRFP43 in modulating HPT axis activity in sheep. The expression of TRH and TSH mRNA and peptides in the hypothalamus and pituitary, as well as plasma concentrations of TSH, free T4 (FT4) and free T3 (FT3) were investigated. Furthermore, it was decided to examine the relationship between QRFP34 activity and mRNA expression of the *Dio1*, *Dio2*, *Dio3* genes in selected tissues of the HPT axis.

## Materials and methods

### Animals and experimental design

All procedures were conducted in accordance with the Code of Ethics of the World Medical Association (Declaration of Helsinki) and the EU Directive 2010/63/EU on animal experimentation. Approval for the study was obtained from the Local Ethics Committee affiliated with the Warsaw University of Life Sciences (no. WAW2/193/2019), in compliance with the Polish Law for the Animal Care and Use of January 21, 2005, and the Polish Law for Animal Protection of September 16, 2011.

Forty-eight female Polish Merino sheep (42-week-old, average body weight = 38.6 ± 3.5 kg) were included in the study. Animals were housed indoors under natural lighting conditions (52°N, 21°E) and fed a standard hay diet with commercial concentrates twice daily, according to the Polish Recommendations for Growing Sheep^20^. The feed was balanced in terms of energy and appropriate nutrients to ensure optimal development of the growing animals. Water and salt licks were available *ad libitum*.

Stainless steel cannulas were surgically implanted directly into the third ventricle (IIIv) of the brain under anaesthesia with atropinum sulfuricum (0.44 mg kg^−1^; Polfa, Warsaw, Poland), ketamine (400 mg per sheep; Vetoquinol Biowet, Gorzów, Poland), and dexmedetomidine (0.05 mg kg^−1^, Dexdomitor^®^; Orion Pharma, Turku, Finland). Permanent stainless steel guide cannulas (ø = 0.8 mm) were inserted into the IIIv at specific coordinates (antero-posterior position ‒ 31 mm, horizontal position ‒ 0.5 mm, mid-sagittal position ‒ 0.10 mm)^21^. The correct placement of the guide cannula was verified by the outflow of upon removal of the guide tube stiletto, as well as by post-mortem examination. After surgery, penicillin-streptomycin (0.1 mL kg^−1^; ScanVet, Gniezno, Poland) and tolfenamic acid (0.05 mL kg^−1^, Tolfine^®^; Vetoquinol Biowet, Gorzów, Poland) were administered *via*subcutaneous injection for four consecutive days, followed by a five-week recovery period. In all experimental animals, oestrus synchronisation was performed 21 days before ICV infusion using Chronogest CR sponges (MSD Animal Health, UK), as described by Przybył (2021)^21^. Animals entered the experiment on day 4–5 after ovulation, coinciding with a decrease in peripheral blood oestrogen levels observed in sheep^22^.

The experiments were conducted from the end of October to the first week of December. Animals were randomly divided into three experimental groups: a control group receiving an intracerebroventricular (ICV) infusion of Ringer-Locke solution (artificial CSF; 480 µL per day; *n* = 16), group I receiving ICV infusion of QRFP43 (Phoenix Pharmaceuticals Inc., USA) at a dose of 10 µg per day (RFa10 group; *n* = 16), and group II receiving QRFP43 at a dose of 50 µg per day (RFa50 group; *n* = 16). Both doses of QRFP43 were diluted in 480 µL of Ringer-Locke solution, and infused at a rate of 120 µL h^−1^. The selection of QRFP43 doses for this experiment was based on information from the literature, as well as our preliminary and previous neuroendocrine experiments^21,23–26^.

During the experiment, 1 h prior to infusion, cannulas were inserted through the guide cannulas and locked in position with the tips positioned approximately 2.0–2.5 mm above the base of the brain; once the tips of the cannulas reached the IIIv, CSF flowed into the infused cannulas. Subsequently, all sheep received four 50-min ICV infusions with 30-min intervals between infusion, performed from 08:40 a.m. to 01:30 p.m. on three consecutive days; the flow rate of the microinjection pump was set at 2 µL min^−1^. Blood samples were collected from animals on Day 3 of infusion every 10 min. from 08:00 a.m. to 01:50 p.m.

After the last infusion, animals were promptly weighed, anaesthetised intravenously using dexmedetomidine (0.05 mL kg^−1^) and pentobarbital (80 ng kg^−1^, Morbital^®^; Vetoquinol Biowet, Poland), and subsequently decapitated. For molecular biological analysis (*n*= 8 + 8 + 8), isolation of the selected hypothalamic structures (area and depth of cuts) was performed according to the coordinates defining the location of individual hypothalamic nuclei described in the stereotaxic atlas of the ovine hypothalamus^27^. Hypothalamic structures from eight sheep from each group (*n*= 8 + 8 + 8) were prepared following the protocols described by Przybył ( 2021)^21^and Szlis (2020)^28^.

## Realtime RT-qPCR

Total mRNA from the medial basal hypothalamus (MBH), anterior pituitary, and thyroid gland was extracted using the NucleoSpin RNA/Protein kit (Macherey-Nagel GmbH & Co., Düren, Germany) according to the manufacturer’s instructions. For complementary DNA (cDNA) synthesis, 1500 ng mL^−1^ mRNA from the selected hypothalamic regions in a total volume of 20 µL was reverse-transcribed using the TranScriba Kit (A&A Biotechnology, Gdynia, Poland) according to the manufacturer’s protocol. Species-specific primers for sheep (*Ovis aries*), determining the expression of housekeeping genes and genes of interest, were designed using Primer 3 software (The Whitehead Institute, Cambridge, MA, USA) and synthesised by Genomed (Warsaw, Poland). The primer sequences are listed in Table 1.

**Table 1:** Primer sequences used in the experiment.

Gene	Primer	Sequence (5’−3’)	Product size (nt)	References
***GAPDH***	Forward	AGAAGGCTGGGGCTCACT	134	^45^
	Reverse	GGCATTGCTGACAATCTTGA		
***PPIC***	Forward	TGGAAAAGTCGTGCCCAAGA	158	^46^
	Reverse	TGCTTATACCACCAGTGCCA		
***ACTB***	Forward	TGGGCATGGAATCCTG	194	^47^
	Reverse	GGCGCGATGATCTTGAT		
***TRH***	Forward	CCTCGTTCGTCAGAGCTG	123	Originally designed XM_012100221.4
	Reverse	CTCGGAGCTGTCTGCGTA		
***TRHR***	Forward	CAGACGCCAAGATGCCTG	150	Originally designed NM_001009407.1
	Reverse	CAGTCGCTGGAGGTCTTC		
***TSHβ***	Forward	GACAGGAAGTGAACTGAACC	184	Originally designed NM_001009410.1
	Reverse	CAGACCACAGATGATGAGC		
***DIO1***	Forward	CCAGGAACCCACACTTCTCC	131	Originally designed XM_004001999.6
	Reverse	CAGTCATGTCCTCCAGTCGC		
***DIO2***	Forward	CAGCGGATGGAACTGAATGC	106	Originally designed JX262388.1
	Reverse	GTCAGCCACGGATGAGAACT		
***DIO3***	Forward	TGGTGCTCAACTTCGGTAGC	216	Originally designed NM_001122650.1
	Reverse	GCTGGAGTTGGTCATCGTGT		

GAPDH – glyceraldehyde-3-phosphate dehydrogenase, ACTB – β-actin, PPIC – peptidylprolyl isomerase C, TRH – thyrotropin-releasing hormone, TRHR – thyrotropin-releasing hormone receptor, TSHβ – thyrotropin subunit beta, DIO1 – deiodinase type 1, DIO2 – deiodinase type 2, DIO3 – deiodinase type 3.

Real-time qPCR was performed using 5× FIREPol EvaGreen qPCR Mix Plus (no ROX; Solis BioDyne, Tartu, Estonia) following the protocol described by Szlis et al. (2018)^29^. Relative gene expression was calculated using the comparative quantitation option implemented in Rotor Gene 6000 software 1.7 (Qiagen GmbH, Hilden, Germany), as well as the Relative Expression Software Tool and the PCR efficiency correction algorithm developed by Pfaffl (2002, 2004)^30,31^. The expression of each investigated gene was normalised to the mean expression of three reference genes, i.e., glyceraldehyde-3-phosphate dehydrogenase (GAPDH), β-actin (ACTB) and peptidylprolyl isomerase C (PPIC). The results are presented in arbitrary units as the ratio of target gene expression to the mean expression of housekeeping genes with the relative gene expression for the group of sheep that received only Ringer-Locke solution infusion set to 1.0 ^28^.

## Immunohistochemical procedures

Hypothalamic sections were incubated for 12 days at 4 °C with primary TRH antiserum (rabbit polyclonal anti-TRH ref. ab216601-100; ABcam, Cambridge, UK), at a dilution of 1:100, following the procedure used in our laboratory^29^, with minor modifications. After incubation with primary antibodies, the sections were incubated for 2 h at room temperature (~ 20 °C) with secondary antibody (IgG H + L fluorescein; Sanofi Diagnostic Pasteur, France) at a dilution of 1:400.

Peroxidase-conjugated antibodies were applied to identify pituitary cells containing TSH. The sections were incubated for 30 min in 2% pre-immune lamb serum, followed by another 30 min incubation in 0.1% hydrogen peroxide, both dissolved in 0.01 M PBS. Next, primary rabbit monoclonal anti-sheep TSHβ antibodies (dilution 1:800; ref. ab155958-100, ABcam, Cambrige, UK) were applied at 4 °C for 4 days. Subsequently, the sections were rinsed in 0.01 M PBS and incubated with secondary antibody (sheep anti-rabbit IgG [H + L] conjugated with peroxidase; ABcam, UK) at a dilution of 1:800 for 2 h at room temperature in 0.1% normal lamb serum in 0.01 M PBS. The colorimetric reaction was initiated by incubating the sections with 0.05% 3′3diaminobenzidine tetrahydrochloride chromogen (Sigma, St. Louis, MO, USA) and 0.001% hydrogen peroxide in 0.05 M Tris buffer.

To assess staining specificity, TRH and TSH antisera were preabsorbed using synthetic TRH (062 − 10, Phoenix Pharmaceuticals INC, USA) and TSHβ (T9265; Merc Life Science, Germany), as described by Szlis (2020)^28^and Wójcik-Gładysz (2019)^23^. No specific cell staining was observed in any of the above controls (data not shown).

Histological analyses of hypothalamic and pituitary sections were conducted using a Nikon type 104 projection microscope (Nikon Corporation, Tokyo, Japan). Analyses were performed using a 4× objective lens for nerve terminals in the median eminence (ME), and a 40× objective lens for the pituitary. Images of immunostained sections were captured using a Panasonic KR222 camera (Matsushita Electric Industrial Co, Osaka, Japan) and displayed on a colour monitor. Images were adjusted for optimal contrast, converted to grey scale (pituitary), fixed at consistent brightness levels, and saved in a buffering system. The area fraction parameter, which indicates the percentage of stained elements in a delineated area, was used for immunoreactive (IR) TRH IR nerve terminals in the ME and IR TSH in the pituitary.

Quantitative analysis was performed within the subareas of interest in each hypothalamus. This involved applying a threshold function to select values that were optically identified as positively stained, while all other values were categorised as unstained. Prior to measurements, images were processed to subtract the background and remove artifacts. The frame size was kept constant for the duration of the image analysis. The area fractions of the immunoproduct in TRH neurons were estimated in every fourth section in the MBH in the delineated area containing the middle part of the ME (~ 20 sections per animal, total area of 100 mm^2^) for each hypothalamus. The area fractions of the TSH immunoproduct were estimated in every 25th section per animal (total field area of 0.1308 mm^2^). Quantitative measurements obtained from each ME and pituitary section were averaged to derive a mean value of each area for each animal.

## TSH, FT4, FT3 radioimmunology

Plasma concentrations of TSH, free FT4 and free FT3 were determined using commercial RIA kits (Beckman Coulter, IMMUNOTECH, Czech Republic) according to the manufacturer’s protocols. The intra- and inter-assay coefficients of variation averaged 3.7% and 8.6% for TSH, 10.29% and 7.58% for FT4 as well as 7.66% and 9.61% for FT3, respectively. The measurement range was 0.04 to 50 mlU/L for TSH, 0.031 to 5.827 ng/100 mL for FT4 and 0.308 to 26.04 pg/100 mL for FT3. The applicability of the aforementioned RIA kits and their specificity for the sheep species were verified during preliminary analyses (unpublished data). The mean concentrations of the tested hormones for individual animals were calculated from the area under the curve, which represents the sum of trapezoidal areas between the curve and the abscissa axis.

### Statistical analysis

GraphPad Prism 5 software (GraphPad Software Inc., La Jolla, CA) was employed for all statistical calculations. All data presented in the graphs represent means ± SEM for each group. Normality of data distribution was tested using the Shapiro-Wilk test. Statistical evaluations of the data were performed using the Kruskal-Wallis test with the post-hoc Dunn multiple comparisons test. Differences resulting in *P* ≤ 0.05 were considered statistically significant.

## Results

### TRH mRNA expression

**Fig. 1:**
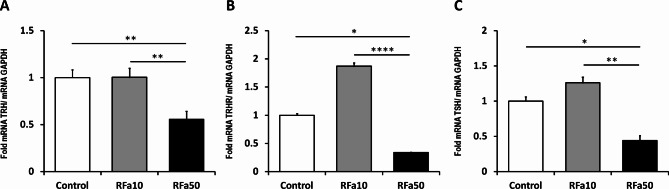
Expression of TRH (**A**) mRNA in the medio-basal hypothalamus (MBH) and expression of TRHR (**B**) and TSHβ (**C**) mRNA in the pituitary. All values are relative to GAPDH mRNA. Data are presented as means ± SEM, with statistical significance denoted as follows: * *P* ≤ 0.05, ** *P* ≤ 0.01, *** *P* ≤ 0.001, **** *P* ≤ 0.00001.

Real-time qPCR analyses demonstrated the presence of TRH mRNA transcripts in the MBH of sheep from all experimental groups. The expression of TRH mRNA was significantly (*P* ≤ 0.01) reduced in the RFa50 group in comparison to control animals (0.56 ± 0.09 and 1.00 ± 0.08, respectively). Similar differences (*P* ≤ 0.01) were observed between the RFa10 and RFa50 groups (1.00 ± 0.09 and 0.56 ± 0.09, respectively). Nevertheless, there were no significant changes observed between the control group and the RFa10 group of animals (Fig. 1A).

## TRHR and TSHβ mRNA expression

At the pituitary level, realtime qPCR analyses revealed the presence of TRHR and TSHβ mRNA transcripts in all experimental groups of sheep. TRHR expression was significantly (*P* ≤ 0.05) lower in the RFa50 group compared to the control group of animals (0.33 ± 0.01 and 1.00 ± 0.03, respectively). Furthermore, statistically significant differences (*P* ≤ 0.0001) were also observed between the RFa10 and RFa50 groups of sheep (1.87 ± 0.06 and 0.33 ± 0.01, respectively). Despite observed higher TRHR mRNA expression in the RFa10 group compared to control animals, no statistical differences were found between these groups (*P* = 0.096) (Fig. 1B).

Simultaneously, a significant (*P* ≤ 0.05) decrease in TSHβ mRNA levels was observed in the RFa50 group compared to the control group (0.44 ± 0.07 and 1.00 ± 0.06, respectively). The expression of TSHβ mRNA was also significantly lower in the RFa50 group than in the RFa10 group (*P* ≤ 0.01, 0.44 ± 0.07 vs. 1.26 ± 0.08, respectively). However, there were no statistical differences between the control group and the RFa10 group (Fig. 1C).

### TRH and TSH immunoreactivity

**Fig. 2:**
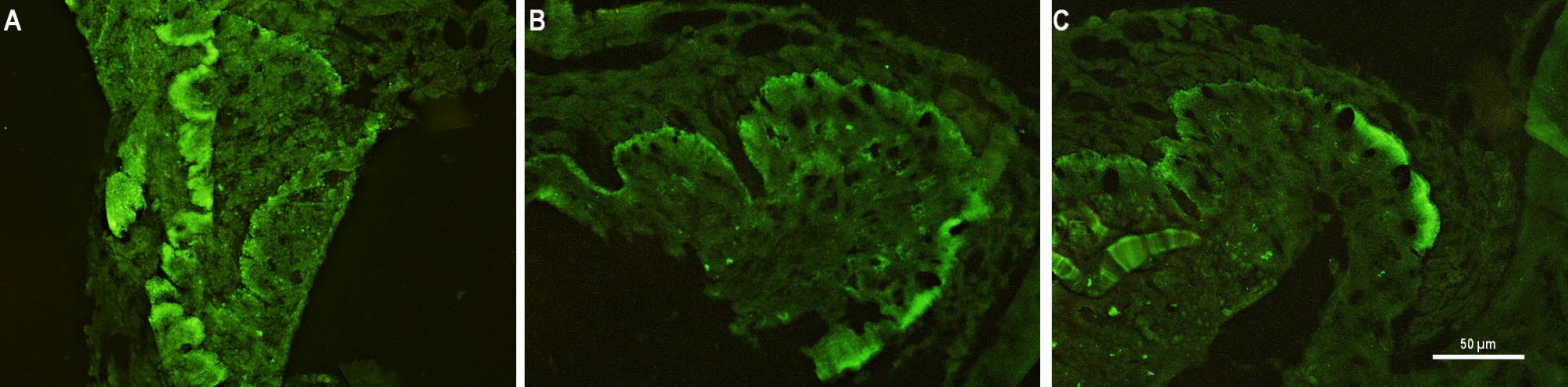
Population of immunoreactive TRH nerve terminals in the median eminence of representative sheep from the control group (**A**), RFa10 group (**B**) and RFa50 group (**C**). Scale bars: 50 μm.

Microscopic observations demonstrated that the locations of immunoreactive (IR) TRH nerves terminals in the ME were similar between the control group and both experimental groups of sheep infused with QRFP43. IR TRH material was predominantly present in the external zone of the ME medial part. Furthermore, infusion of QRFP43 in both the RFa10 and RFa50 groups did not alter the amount of IR TRH material in the ME (5.08 ± 0.05% and 5.03 ± 0.07%, respectively) compared to the control group (5.12 ± 0.05%; Fig. 2).

**Fig. 3:**
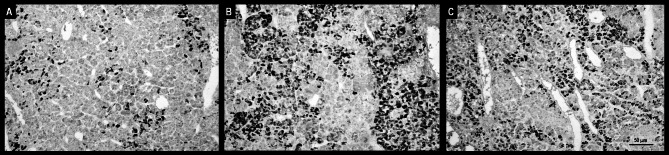
Populations of immunoreactive TSH pituitary cells from the control (**A**), RFa10 (**B**) and RFa50 (**C**) groups. Scale bars: (**A–C**) 50 μm.

Visible differences in IR TSH pituitary cells between the control group and both experimental groups of sheep receiving QRFP43 were noted. The number of visible TSH-positive stained cells and the intensity of immunostaining were higher in the RFa10 and RFa50 groups of sheep compared to control animals. These observations were confirmed by the measurements of the percentage area occupied by IR TSH material, which showed a significant increase (*P* ≤ 0.01) in IR material in the RFa10 and RFa50 sheep groups (13.25 ± 0.13% and 13.90 ± 0.11%, respectively) compared to the control group of animals (6.03 ± 0.10%; Fig. 3).

### TSH, FT4 and FT3 blood levels

**Fig. 4:**
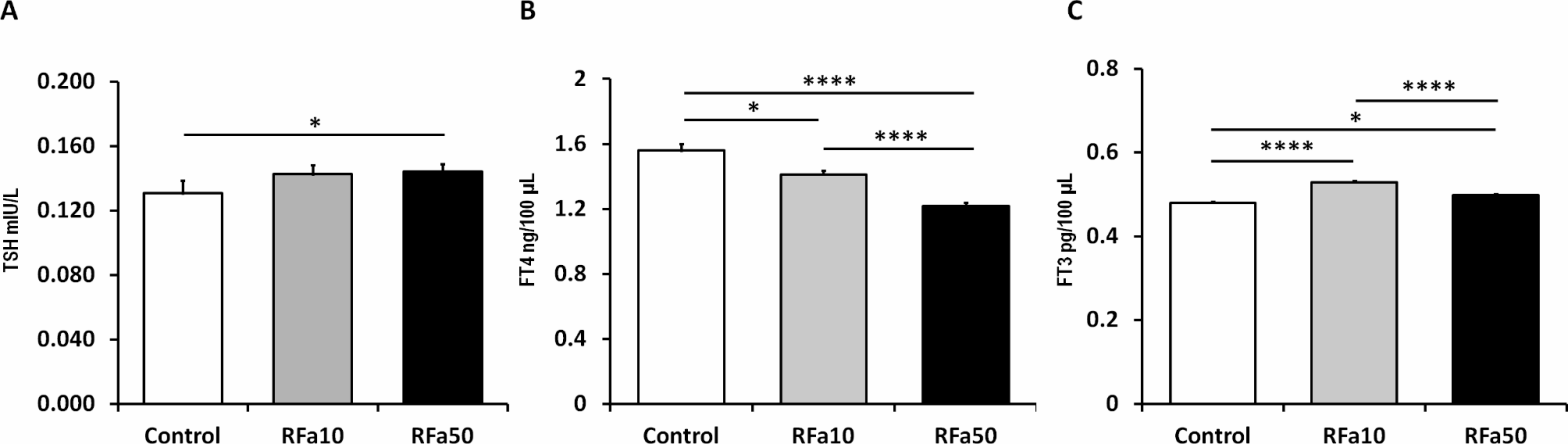
Concentrations of TRH (**A**), FT4 (**B**) and FT3 (**C**) in blood plasma. Data are presented as means ± standard error of measurement. Statistical significance is denoted as follows: at * *P* ≤ 0.05, ** *P* ≤ 0.01, *** *P* ≤ 0.001, **** *P* ≤ 0.00001.

The basal mean plasma TSH concentration in the control group of sheep was 0.131 ± 0.008 mlU/L. The mean plasma TSH levels increased in both the RFa10 (0.143 ± 0.006 mlU/L) and RFa50 (144 ± 0.004 mlU/L) groups of sheep; however, statistically significant was only the difference between the RFa50 group and the control group (*P* ≤ 0.01; Fig. 4A). Moreover, a significant decrease in FT4 concentrations was observed between the control and RFa10 groups (*P* ≤ 0.01; 1.56 ± 0.04 and 1.41 ± 0.02 ng/100 µL, respectively), the control and RFa50 groups (*P* ≤ 0.0001, 1.56 ± 0.04 and 1.22 ± 0.02 ng/100 µL, respectively), as well as between the RFa10 and RFa50 groups of animals (*P* ≤ 0.0001) (Fig. 4B). Additionally, a significant increase in FT3 concentration was recorded between the control and RFa10 groups (*P* ≤ 0.0001, 0.48 ± 0.003 and 0.53 ± 0.003 pg/100 µL, respectively), the control and RFa50 groups (*P* ≤ 0.01, 0.48 ± 0.003 and 0.50 ± 0.003 pg/100 µL, respectively), and between the RFa10 and RFa50 groups of sheep (*P* ≤ 0.0001) (Fig. 4C).

### Dio1, Dio2 and Dio3 mRNA expression

**Fig. 5:**
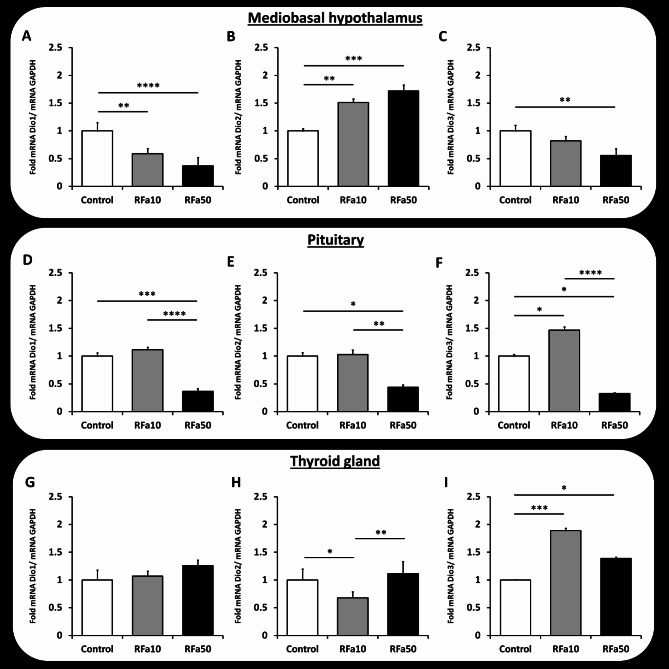
Expression of DIO1 (**A, D, G**), DIO2 (**B, E, H**) and DIO3 (**C, F, I**) mRNA in the medio-basal hypothalamus (MBH; **A, B, C**), pituitary (PIT; **D, E, F**) and thyroid gland (Th; **G, H, I**). All values are relative to GAPDH mRNA. Data are presented as means ± SEM, with statistical significance denoted as follows: * *P* ≤ 0.05, ** *P* ≤ 0.01, *** *P* ≤ 0.001, **** *P* ≤ 0.00001.

Realtime qPCR analyses showed that Dio1, Dio2 and Dio3 mRNA transcripts were present in the MBH, pituitary and thyroid gland of sheep from all experimental groups. A significant decrease in mRNA Dio1 expression was observed in the RFa10 (*P* ≤ 0.01, 0.59 ± 0.09) and RFa50 (*P* ≤ 0.0001, 0.37 ± 0.15) groups of sheep compared to control animals (1.00 ± 0.15). There were no statistical differences between the experimental groups of animals receiving QRFP43 infusion (Fig. 5A). Furthermore, a significant increase (*P* ≤ 0.01) in Dio2 mRNA expression was observed between the control and RFa10 groups (1.00 ± 0.04 and 1.50 ± 0.06, respectively). A similar significant increase (*P* ≤ 0.001) was also observed between the control group and the RFa50 group (1.00 ± 0.04 and 1.72 ± 0.11, respectively). No differences between the RFa10 and RFa50 groups of sheep were recorded (Fig. 5B). Dio3 mRNA expression was significantly (*P* ≤ 0.01) lower in the RFa50 group in comparison to the control group of animals (0.56 ± 0.13 and 1.00 ± 0.10, respectively, Fig. 5C).

In the pituitary, Dio1 mRNA expression was significantly (*P* ≤ 0.001) lower in the RFa50 group compared to the control group of sheep (0.36 ± 0.05 and 1.00 ± 0.06, respectively). A significant (*P* ≤ 0.0001) difference was also observed between the RFa10 and RFa50 groups (1.10 ± 0.04 and 0.36 ± 0.05, respectively, Fig. 5D). Furthermore, a significant decrease (*P* ≤ 0.05) in Dio2 mRNA expression was observed in the RFa50 group compared to control animals (0.44 ± 0.04 and 1.00 ± 0.06, respectively, Fig. 5E). Regarding Dio3 mRNA expression, in comparison to control animals, the RFa10 group had a significantly higher levels of this transcript (*P* ≤ 0.05) (1.00 ± 0.08 and 1.47 ± 0.04, respectively), while statistically lower levels (*P* ≤ 0.05) were recorded in the RFa50 group (1.00 ± 0.08 and 0.33 ± 0.02, respectively, Fig. 5F).

In the thyroid gland, Dio2 mRNA expression was significantly (*P* ≤ 0.05) lower in the RFa10 group than in control animals (0.68 ± 0.11 and 1.00 ± 0.20, respectively). Moreover, in comparison to the RFa10 group, a significant increase (*P* ≤ 0.01) in this transcript levels were observed in the RFa50 group (0.68 ± 0.11 and 1.11 ± 0.22, respectively; Fig. 5H). The concentration of Dio3 mRNA transcripts was significantly higher in the RFa10 group (*P* ≤ 0.001, 1.89 ± 0.04), as well as in the RFa50 group (*P* ≤ 0.05, 1.39 ± 0.02) compared to the control group of sheep (1.00 ± 0.01; Fig. 5I). However, no differences were observed in Dio1 mRNA expression in the thyroid gland (Fig. 5G).

## Discussion

Following its initial identification, QRFP43 has become a focal point of extensive research, particularly regarding its role in food intake regulation. Many studies have suggested that this peptide may induce orexigenic effects in rodents, leading to increased food consumption and subsequent weight gain. At the same time, it has been suggested that the effect of QRFP43 on the body’s energy homeostasis may depend on a number of factors. Research indicates that these variables could include type of diet, caloric intake, QRFP43 administration method, hormonal status of the animals or even the specific animal model utilised in experiments^32–34^. Regulation of the body’s energy equilibrium is recognised as a highly complicated process involving many systems, hormones and neurotransmitters. The HPT axis, besides the satiety and hunger centre situated in the hypothalamus, stands as a pivotal regulator of energy status, directly and indirectly influencing metabolic regulation in all cells of the body. Consequently, conducting studies aimed at elucidating how QRFP43 can affect the secretory activity of the HPT axis are of great importance.

The present study investigated the impact of QRFP43 on the expression and secretion of key hormones of the HPT axis in sheep. The findings indicated that centrally administered QRFP43 led to a decrease in TRH mRNA expression in the MBH, although it did not alter TRH protein expression in the ME nerve terminals. These results represent novel insights, as they are the first to demonstrate a potential effect of QRFP43 on mRNA expression in the hypothalamic region associated with the HPT axis. Considering orexigenic properties of QRFP43, one might anticipate a stimulatory effect on TRH synthesis and secretion in the hypothalamus, resulting in increased metabolism or energy storage. Despite the observed lack of differences in the amount of IR TRH material in the ME, a reduction in TRHR mRNA expression was recorded at the pituitary level. These results may suggest a diminished sensitivity of this organ to the stimulatory signals of TRH.

In addition, QRFP43 was observed to cause a decrease in TSHβ mRNA expression in the pituitary; however, simultaneously, elevated quantities of IR TSH materials were also observed in pituitary cells. This discrepancy between the expression at mRNA and protein levels in in vivo research are not uncommon and can be attributed to post-transcriptional modulation of mRNA processes. Our previous studies have demonstrated similar phenomena, where hormones such as leptin, ghrelin, obestatin, or brain-derived neurotrophic factor could also differentially affect mRNA and protein expression of various hormones associated with the somatotrophic and gonadotrophic axes in sheep^21,28,35^.

The results obtained in the pituitary regarding TSH can be interpreted as an increase in the secretion of this hormone, a notion supported by the results of the radioimmunoassay of plasma TSH levels. In the present study, a significant elevation in TSH levels was observed in animals following QRFP43 infusion, indicating enhanced release from pituitary cells. Interestingly, a study conducted on male rats with hypothyroidism showed that kisspeptin, another hormone belonging to the RF-amide family, did not affect TSHβ mRNA expression in pituitary cells^36^. Similarly, investigations in male rhesus monkeys indicated that kisspeptin exerted no effect on plasma TSH concentrations in these animals^37^. Corresponding findings were reported in human studies, which found no correlation between circulating levels of kisspeptin and TSH concentration; additionally, no interaction was observed between long-term kisspeptin administration and TSH levels in those patients^38,39^. Given these insights, it can be assumed that different groups of RF-amides exert varying effects on TSH expression, and their impact may vary among individual animal species.

Our analyses also showed that ICV administration of QRFP43 decreased FT4 concentrations, while simultaneously increasing plasma FT3 levels. These observations indicate that QRFP43 may stimulate metabolism and energy expenditure in sheep by increasing the availability of T3 in the bloodstream. Interestingly, Hasan^40^reported a positive correlation between T4 and kisspeptin concentrations in the peripheral blood of women suffering from hyperthyroidism. In addition, a study in young female patients with betathalassemia showed a positive correlation between kisspeptin concentration and T3 levels in blood^41^.

To the best of our knowledge, these findings represent the first evidence of QRFP43 effects on the functioning of the HPT axis across all its organisational levels in vivo. The absence of comparative data for reliable contextualisation of our results suggests a further direction of research that should focus on testing the role of this RF-amide in other animal species. Further investigations are also needed to elucidate the interactions of QRFP43 with the feedback loops of the HPT axis.

Another aspect addressed in the current study was the potential modulation by QRFP43 of mRNA expression of genes coding for deiodinase enzymes, including DIO1, DIO2, and DIO3, in selected tissues of the HPT axis (ventromedial hypothalamus, pituitary and thyroid gland). Considering that the half-life of T4 in the human body is approximately one week, DIO activity in tissues has a significant impact on T3 availability^42^. The results showed that QRFP43 reduced the expression of DIO1 and DIO3 mRNA in the sheep MBH and increased the expression of DIO2 mRNA in this structure. Increased levels of DIO2 have been associated with enhanced conversion of T4 into T3 by cells. Studies conducted on Siberian hamsters have further elucidated this relationship, showing that animals exposed to prolonged periods of light (long day) had increased DIO 2 and decreased DIO3 levels in the brain. These changes led to increased availability of active T3 in brain tissues^43,44^.

The available literature lacks comprehensive data on the expression profiles of DIO in tissues forming the HPT axis. Our analyses have provided novel insights by demonstrating, for the first time, significant alterations in DIO expression in animals administered with QRFP43. Specifically, our findings revealed a significant reduction in DIO1 and DIO2 mRNA expression in pituitary cells, albeit solely at the higher dose of QRFP43. As regards DIO3, observations indicate that the lower dose administered in the experiment stimulated mRNA expression of this gene, while the higher dose elicited the opposite effect, leading to decreased DIO3 mRNA expression. These results suggest that it is the dose of peptide administered to the animals that represent another factor influencing the ultimate effect of RF-amides.

At the thyroid level, no discernible effect was observed of QRFP43 on DIO1 mRNA expression. QRFP43 at a dose of 10 µg caused a decrease in the expression of DIO2 transcript, while the higher dose of 50 µg resulted in increased transcription of this gene. Conversely, for DIO3, both doses used in the experiment led to elevated expression of DIO3 mRNA. These findings suggest a potential shift in thyroid cell metabolism towards reducing the production of the active form of T3, while promoting the formation of the inactive form of rT3. Moreover, similar to the observations in the pituitary gland, these results emphasise the fact that the effect of RF-amides is strictly dependent on the concentration/dose of these peptides.

**Fig. 6:**
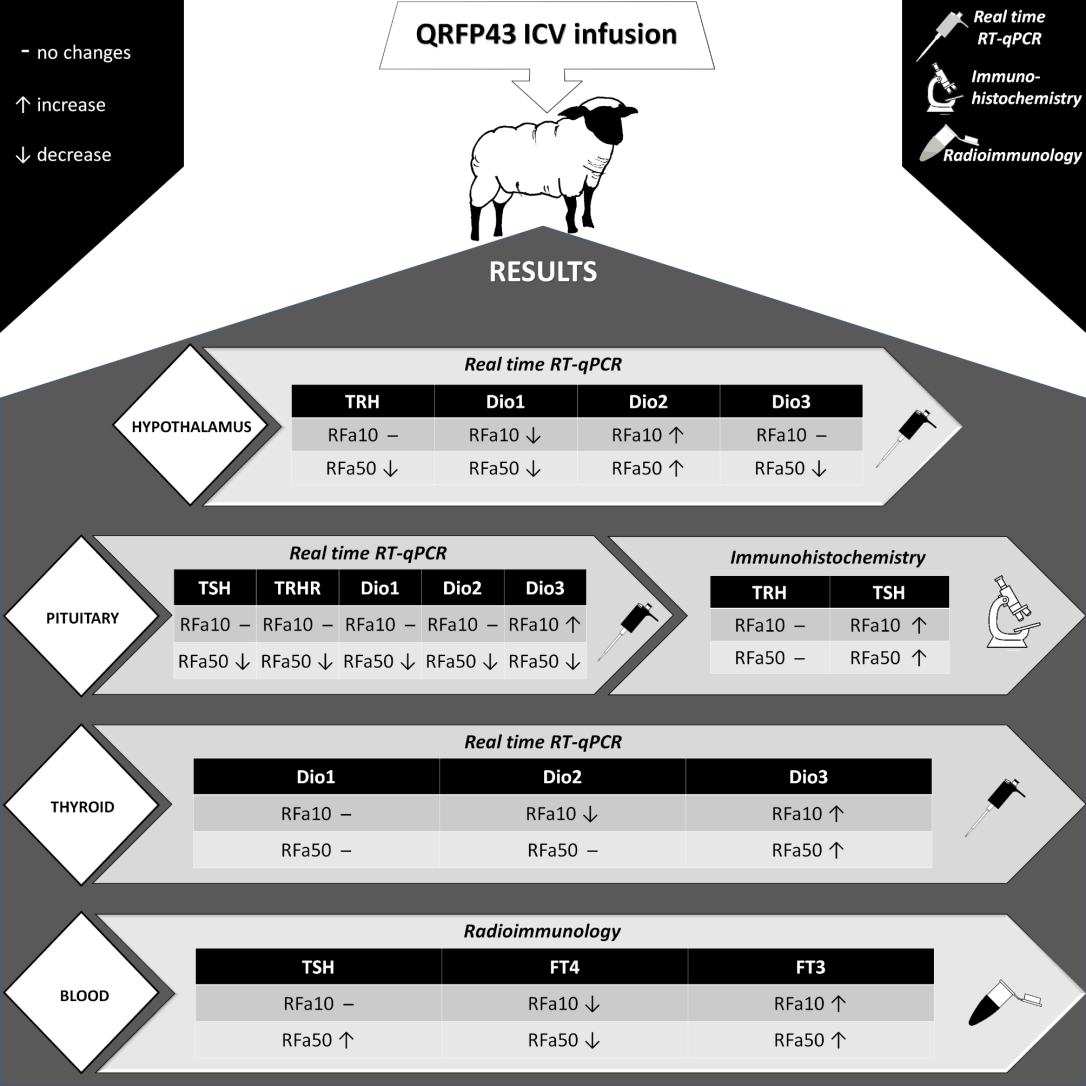
Graphical summary of the obtained results.

Our experiment indicated that QRFP43 stimulated the HPT axis, which was particularly evident in the levels of the TSH hormone, as well as FT4 and FT3 in peripheral blood. It should be noted that a three-day infusion of QRFP43 led to a reduction in the expression of certain HPT axis genes (Fig. 6). This observation suggests a potential gradual saturation of tissues with the protein, triggering mechanisms aimed at suppressing axis activity to maintain homeostasis. Therefore, it seems necessary to conduct further research that could clarify the precise mechanism of action of this RF-amide in the body and unravel its short and long-term effects. Summarising the results of the present experiment, it can be concluded that QRFP43 modulates the secretory activity of the HPT axis at all levels of its organisation. Furthermore, QRFP43 alters the mRNA expression profiles of DIO1, DIO2 and DIO3 in the tissues of glands forming this axis, leading to discrete changes in the metabolism of the examined cells and their response to signals transmitted by T4 and T3.

## Electronic supplementary material

Below is the link to the electronic supplementary material.

Supplementary Material 1

## Data Availability

All data generated or analysed during this study are included in this published article (and its Supplementary Information files) and are also available from the corresponding author on reasonable request.

## Abbreviations

IIIv: The third ventricle
CSF: Cerebrospinal fluid
Dio1: Iodothyronine deiodinase type 1
Dio2: Iodothyronine deiodinase type 2
Dio3: Iodothyronine deiodinase type 3
FT3: Free triiodothyronine
FT4: Free thyroxine
HPG: The hypothalamus–pituitary–gonadal axis
HPT: The hypothalamic–pituitary–thyroid axis
ICV: Intracerebroventricular
LHA: The lateral hypothalamic area
MBH: The mediobasal hypothalamus
PVN: The paraventricular nucleus
T3: Triiodothyronine
T4: Thyroxine
TRH: Thyrotropin–releasing hormone
TSH: Thyrotropin
VMH: The ventromedial hypothalamus

## Declarations

### Software used in experiments

1: Primer3web, version 4.1.0; The Whitehead Institute, Cambridge, MA; https://primer3.ut.ee/. 2: The Rotor Gene 6000 software 1.7 containing Relative Expression Software (Qiagen GmbH, Hilden, Germany); software provided by the manufacturer. 3: Best-Keeper software; http://www.gene-quantification.de/bestkeeper.html. 4: GraphPad Prism 5 (GraphPad Software Inc., La Jolla, CA); https://www.graphpad.com/features.
